# Navigating fragmented services: a gender-based violence (GBV) critical feminist analysis of women’s experiences engaging with health and social supports in three Canadian cities

**DOI:** 10.1186/s12889-025-21919-w

**Published:** 2025-03-31

**Authors:** Katherine Rudzinski, Lara F. Hudspith, Adrian Guta, Scott Comber, Linda Dewar, Wendy Leiper, Kim Hawkins, Lady Laforet, Rajwant Raji Mangat, Phoebe M. Long, Ingrid Handlovsky, Vicky Bungay

**Affiliations:** 1 Capacity: The Centre for Research in Community Engagement and Gender Equity, School of Nursing, University of British Columbia, T201-2211 Wesbrook Mall, Vancouver, BC V6T 2B5 Canada; 2 School of Social Work, University of Windsor, 167 Ferry Street, Windsor, ON N9A 0C5 Canada; 3 Rowe School of Business, Dalhousie University, 6100 University Ave, PO BOX 15000, Halifax, NS B3H 4R2 Canada; 4 Inner-City Women’s Initiatives Society, 101 E Cordova St, Vancouver, BC V6A 1K7 Canada; 5 Welcome Centre Shelter, 500 Tuscarora St, Windsor, ON N9A 3M2 Canada; 6 West Coast Women’s Legal Education and Action Fund (LEAF), 409 Granville St, Vancouver, BC V6C 1T2 Canada; 7 School of Nursing, University of Victoria, 3800 Finnerty Road, HSD Building A402a, Victoria, BC V8P 5C2 Canada

**Keywords:** Gender-based violence, Women, Complex needs, Poverty, Service access, Health and social care

## Abstract

**Background:**

Gender-based violence (GBV) remains a pervasive public health crisis with devastating impacts on women’s health and well-being. Women experiencing GBV face considerable barriers accessing appropriate and timely health and social services. This study explored women’s experiences with health and social services in three Canadian cities to understand critical challenges and strengths in service provision for women experiencing GBV.

**Methods:**

In-depth interviews were conducted with self-identifying women (*n* = 21) who had accessed health or social care services and with service providers (*n* = 25) in three Canadian cities between February 2021 and November 2022. Women’s interviews focused on experiences engaging with services including what worked well, the challenges they faced, and their recommendations to enhance service delivery to women experiencing violence. Staff interviews focused on their experiences of providing services within their organization, and the strengths and challenges in providing services to women within their community. Data were analyzed using reflexive thematic analysis with a gender-based violence critical feminist lens.

**Results:**

We organized the findings into three interrelated themes. First our results show how the systems within which health and social services are organized, are not designed to meet women’s complex needs, with rigid structures, siloed services, and stigmatizing cultures creating significant barriers. Second, the data illustrate how service providers support and empower women through practices such as providing key information, assisting with administrative tasks, offering material resources, and addressing discrimination through advocacy and accompaniment. Third, our findings demonstrate how building an effective working relationship characterized by trust, non-judgment, and collaboration is crucial for service engagement and women’s overall well-being.

**Conclusions:**

Findings illuminate critical public health challenges as women navigate fragmented services across multiple and siloed systems not designed to meet their complex needs. There is an urgent need for systemic change to create more integrated, responsive support systems for women experiencing GBV. This includes addressing underlying structures perpetuating gender inequities and violence. Facilitating safe access to holistic services that consider women’s preferences is crucial. Effective working relationships built on trust, respect, and power-sharing are key to supporting women’s agency and addressing their interconnected needs.

**Supplementary Information:**

The online version contains supplementary material available at 10.1186/s12889-025-21919-w.

## Background

Gender-based violence (GBV) remains a pervasive global public health crisis with devastating impacts for women, families, communities, and society. GBV encompasses harmful threats, acts, practices and policies directed toward an individual, group, or community based on their actual or perceived gender identity and/or expression [1–4]. While people of all genders experience violence, women, inclusive of trans women^1^, experience a disproportionate burden of GBV [5, 7–9]. GBV has devastating impacts on women’s health and well-being including, psychological trauma, sexual and reproductive health problems, physical injuries, traumatic brain injury, increased HIV risk, deprivation of human rights, and premature death [5, 8–18]. Broader impacts of GBV include disrupted families, childhood trauma, women’s reduced participation in society, and global economic costs estimated at 1.5 trillion USD annually (2% of GDP) through lost productivity and increased healthcare and legal expenses [5, 10, 19, 20]. In light of this, the World Health Organization (WHO) has urged the health sector to “provide comprehensive health care to women subjected to violence” [20], while the United Nations has called for “women and girls’ access to quality, multi-sectoral services essential for their safety, protection and recovery [from violence]” [21].

GBV is rooted in patriarchal power relations and is unequally experienced, due to prevailing social and economic inequities [1, 2, 5, 6, 10, 22]. While often reduced to physical acts of violence (e.g., intimate partner violence (IPV), sexual and/or physical assault; [2, 23]), GBV manifests across a broad spectrum, encompassing interpersonal violence (e.g., physical, sexual, psychological, economic acts of harm; including threats of violence, harassment, coercion) and structural violence through unjust policies and institutional practices that create conditions for interpersonal violence and normalize violence against women [2, 6, 24]. GBV is multifactorial in nature, emerging from multiple, interconnected societal, economic, cultural, and institutional factors that work together to perpetuate harm, yet it is fundamentally shaped by systemic gender inequities. Understanding GBV requires examining the intersection of gender with other axes of power and privilege (e.g., race, social class, colonial histories, sexuality, (dis)ability, age) [25]. Different aspects of women’s social location shape how they are treated by others and institutions and impact the level, severity, and types of oppression they face and how they experience GBV. For instance, Indigenous women, racialized women, trans women, women with disabilities, younger women, and women living in poverty are disproportionately affected by GBV as a direct result of historical and ongoing colonization, systemic racism, and ableism prevalent in society [11, 12, 26].

GBV remains a serious public health issue in Canada Annually 102 women and girls are killed in GBV incidents across the country with over 90% of perpetrators being intimate partners or family members of victims [27]. The economic impact of spousal violence alone costs Canada approximately $7.4 billion yearly [28]. Despite these figures, GBV is severely underreported due to fears of stigma, victim-blaming, and retaliation, with only 6% of sexual assaults reported to police [29–32]. Indigenous women face disproportionately high rates of sexual violence, being 3 times more likely than non-Indigenous women to experience this form of violence [33].

Women experiencing GBV face considerable barriers accessing appropriate and timely health and social services commensurate with their needs [34–37]. These barriers include availability issues (e.g., prolonged wait times, limited hours of operation), accessibility challenges (e.g., geographical barriers, lack of transportation or childcare), and economic hardships that make engagement difficult for those living under conditions of chronic or episodic poverty [35, 38, 39]. While GBV transcends socioeconomic strata, it is crucial to recognize that economic precarity creates additional barriers to both disclosure and help-seeking. GBV stigma contributes to disrespectful, unsafe, and inequitable service experiences, creating mistrust in care and the potential for re-traumatization (e.g., violence experienced through policies that negate women’s agency, reproduction of damaging power relationships between providers and women) [35, 40–43]. Moreover, the siloed nature of services fails to address women’s complex needs [5, 29, 30]. Such marginalizing conditions create an environment where women may be reluctant to access supports, delay help-seeking, and are often unable to access care until crisis occurs, increasing health risks and perpetuating social inequities [36, 44].

While women experiencing GBV are resourceful in managing their health and well-being [2, 45], there is increasing demand for inclusive program design that takes a strengths-based approach and is responsive to women’s actual (not perceived) needs. Despite the growth in women-specific services and women-centred care, including substantial investment in programs for women survivors of IPV (e.g., crisis hotlines, victim-advocacy support groups, financial literacy programs, emergency shelters; [46–50]), women are often forced to rely on an ineffective patchwork of services organized across multiple systems. This fragmentation not only disadvantages women, making them appear disconnected from health and social service agencies, but also serves to “other” experiences of GBV, placing them as women’s only concerns, or addressable only through survivor-focused interventions. Social determinants of health, such as income, education, housing, and social support networks, significantly influence women’s experiences of GBV and their ability to access and benefit from support services, either exacerbating vulnerabilities or providing protective effects, underscoring the need for holistic, intersectional approaches for addressing GBV. It is, therefore, urgent, timely, and essential that we learn from women’s direct experiences with these services, in order to elucidate what is required to optimize services to better meet their needs.

A critical feminist lens offers a valuable framework for examining women’s experiences with health and social services in the context of GBV. This approach allows us to recognize that although GBV is common, these occurrences are not innate – they are rooted in power relations and inequities, specifically in a binary and hierarchical system of gender that supports inequality, and perpetuates patriarchy, sexism, and damaging gender stereotypes [2, 51–53]. Within patriarchal society, social, legal, financial, and political frameworks maintain male dominance [51, 54], constructing ‘masculine’ and ‘feminine’ as polar opposites, with feminine being subordinate, and upholding strict binary gender norms. This critical feminist lens enables us to question the status quo, considering the social context in which GBV occurs in order to understand the broader function of this form of violence (e.g., to humiliate, to coerce, to control) as well as the conditions under which such oppression is possible, or in some cases seen as acceptable [51, 54, 55]. This perspective helps reveal how multiple forms of oppression shape women’s interactions with services within these larger structures of patriarchal power and control. Moreover, this lens also allows us to find potential ways to challenge and eradicate these structures in order to better support women’s health and well-being. The scholarly discourse surrounding gender and GBV encompasses diverse theoretical frameworks and epistemological traditions. Our approach, while grounded in critical feminist theory, acknowledges and engages with the multifactorial nature of GBV. Using a GBV critical feminist approach, this paper explores self-identifying women’s experiences with health and social services, in three Canadian cities. This study aims to better understand the critical challenges women face when engaging with these services and identify what they perceive as strengths in current service provision. This understanding is crucial for developing respectful, trauma-informed, and strengths-based services for women experiencing GBV, while identifying the broader systemic changes needed to make such service design, implementation and provision a reality.

## Methods

The data used in this paper were drawn from a larger 7-year, community-based research study examining the adaptation of a socially complex outreach intervention with varied sub-groups of women experiencing GBV in partnership with a range of non-profit women serving organizations [2]. Research participants were self-identifying women (inclusive of cis and trans women). The organizations ranged from emergency and short-term shelter, outreach and health promotion, legal services, and victim specific services including support with police and health care engagement, counselling, and criminal justice proceedings. Two organizations were specific to self-identifying women and one served people of all genders although women represented the vast majority of their clientele. Adaptation included tailoring the intervention to the local context of three different urban cities in Canada (one metropolitan centre, one mid-sized city, and one small urban area) and community-service organization partners focused on providing various supports including housing, legal, and victim services. As each city had unique services available and local norms concerning service delivery to women affected by GBV, it was essential that we develop a nuanced understanding of each local context. Specifically, we strove to understand the contexts surrounding women’s service utilization and needs, as well as the strengths and challenges of each site and participating organization in providing services and supporting women to access other, external services. To aid in understanding the local context and the unique features that needed to be considered for adaptation of the intervention prior to implementation we undertook in-depth interviews with women (*n* = 21) attending to services within their community and community service staff and managers (*n* = 25) that provided such services. The interview guides utilized (see Supplementary File 1) were developed for the sole purpose of the study. Interviews were carried out in the context of post-lockdown COVID-19, and so capture the additional challenges present in that context. The data collected during these interviews serve as the data for this paper. This research was conducted in accordance with The Tri-Council Policy Statement: Ethical Conduct for Research Involving Humans (TCPS 2) and the Declaration of Helsinki. Ethical approval for this research was obtained from the University of British Columbia (UBC) Behavioural Ethics Board (Ethics Certificate Numbers: #H20-02864, #H21- 01301) and University of Windsor Research Ethics Board (Ethics Certificate Number: #21-105).

With the aid of the not-for-profit community service staff, women were invited to participate if they were 18 years of age or older, able to communicate verbally in English, and had accessed health or social care services within the not-for-profit sector within their community in the previous five years (e.g., doctors, counselling etc.). Partnering organizations’ staff and managers were invited to participate by members of the research team. Interviews occurred from February 2021 to November 2022. All interviews were conducted by trained research staff members and occurred in person or via zoom depending on the participant’s preference. Consent was obtained verbally and all participants received incentives in keeping with research practices within their communities. Women received $30 in cash, and service providers were offered a $25 gift card. Transcripts were audio recorded, transcribed and checked for accuracy by members of the research team.

Most of the participants identified as cis-women (90.5% of women; 100% of service providers), and the majority identified as White (42.9% of women; 76.9% of service providers). The ages of the women ranged from 20 to 57, with an average age of 38.1, and the ages of the service providers ranged from 24 to 65, with an average age of 41.2. Please see Tables 1 and 2 for further participant characteristics. Women’s interviews focused on their experiences of engaging with the partnering organizations including what worked well and the challenges they faced. Women were also asked specifically about their recommendations to enhance service delivery to women experiencing violence. Staff and managers were similarly asked about their experiences of providing health and social care services within their organization including the process of referral to other services, and the strengths and challenges in providing services to women within their community. Throughout this paper we privilege data gathered through women’s interviews, as our focus was to learn more from women and their experiences engaging with services. We use service provider interviews as a complement to women’s voices, and as a way of providing additional context, especially when providers are able to speak to their specific practices and empowering strategies used to support women and establish strong working relationships with the women they serve.

### Data analysis

Interview transcripts were analyzed using Reflexive Thematic Analysis [56]. First, two members of the research team (KR and LH) read (and re-read) the transcripts to inductively develop individualized initial coding schemes for the women’s transcripts and service provider’s transcripts respectively. These coding schemes were then shared with other research team members (VB and PL) for comment and inputted into NVivo R qualitative data software to refine and complete team coding. Initially, KR and LH applied the refined coding schemes to a subset of transcripts (*N* = 12). The final codes were then grouped together based on broader patterns of meaning, into major themes. Applying a GBV critical feminist lens to the data, we examined women’s discussions of challenges accessing services paying particular attention to how these services were organized and provided across multiple and siloed systems (e.g., housing, health, social, criminal legal, welfare). Data were further coded to detail women’s perspectives on the strengths of existing services providing care for women experiencing GBV. The themes were refined via discussion with the research team, including periodically returning to raw data to ensure consistency with women’s reported strengths and challenges of accessing services. Final analysis to identify core themes included discussion with the entire team. In reporting these findings, we attribute quotes to women, as service users, and service providers using the following identification scheme [W#] and [SP#], respectively.

**Table 1:** Women’s characteristics

	*n* = 21 (%)
**Gender Identity**	
Cis-woman Transgender woman Non-binary and gender fluid	19 (90.5) 1 (4.8) 1 (4.8)
**Ethnicity**	
White Indigenous Black Multiple ethnicities	9 (42.9) 6 (28.6) 2 (9.5) 4 (19.1)
**Age**	
20–29 30–39 40–49 50–59	3 (14.3) 10 (47.6) 6 (28.6) 2 (9.5)
**Education**	
High School – pre-Grade 12 High School – Diploma Trade Post-Secondary Degree Some College College – Bachelors	5 (23.8) 11 (52.4) 1 (4.8) 2 (9.5) 2 (9.5)
**Difficulty living on current income**	
1 (Not at all difficult) 2 3 4 5 (Impossible)	0 (0.0) 2 (9.5) 5 (23.8) 3 (14.2) 11 (52.4)

**Table 2:** Service provider characteristics

	*n* = 25 (%)
**Gender Identity**	
Cis-woman	25 (100.0)
**Ethnicity**	
White Multiple ethnicities South American South Asian	20 (76.9) 4 (15.4) 1 (3.9) 1 (3.9)
**Age**	
20–29 30–39 40–49 50–59 60–69	4 (17.4) 6 (26.1) 5 (21.7) 3 (13) 5 (21.7)
**Education**	
High School – pre-Grade 12 High School – Diploma Trade Post-Secondary Degree Some College College – Bachelors Masters	0 (0.0) 2 (7.7) 0 (0.0) 0 (0.0) 15 (57.7) 9 (34.6)
**Professional Licensure** (e.g., Registered Social Worker, Registered Counsellor)	
Yes No	8 (30.8) 18 (69.2)
**Average number of years in current role (range in years)**	4.7 (0.08–20)

## Results

Although not specifically asked about, the women talked at length about their experience(s) of GBV including IPV, assaults, trauma and abuse, and the related and often consequential and intersecting complexities in their daily lives. For instance, many women were living under conditions of chronic poverty, while most, if not all, had experienced periods of episodic severe material deprivation. Women’s basic needs such as safe, appropriate, and accessible housing, financial resources, and food were often unmet. Many women also experienced various physical and mental health struggles, substance use issues, legal challenges, including experiences of incarceration and child custody issues. Many experienced “crises” for short or extended, often reoccurring, periods. Some women found themselves “in crisis” because they were simultaneously experiencing a number of the aforementioned complexities, while for others this centered on specific urgent situations that they faced, such as suicidality or other mental health emergencies, housing loss, or substance use problems:

*“I felt like … after the assault and you know*,* kind of being … left behind with this debt and these bills you know*,* it felt like I was starting to get really hopeless and I kind of slipped into a depression for a while*,* right. Plus*,* I had a broken tailbone*,* so I was stuck in bed.”* [W11].

As women discussed their daily challenges, seeking services was an essential aspect of attempting to address their ongoing health and well-being needs. Women noted numerous barriers to care, a situation exacerbated by inaccessible information about available resources as well as organizational processes of eligibility, accessibility, and discrimination that compounded their difficulties. Women noted what “good” engagement looked like and the characteristics of staff and features of organizations that contributed to their success. In the subsequent presentation of findings, we organize women’s experiences into three interrelated themes that demonstrate: key challenges regarding services provided within systems not designed to meet women’s complex needs; specific practices and empowering strategies used by providers to support women; and underlying approaches to building an effective working relationship crucial for service provision.

### Theme one: systems are not designed to meet women’s complex needs

It was evident that the legal, social, and health care service systems (hereafter referred to as systems) providing supports for women had several design flaws that limited their capacity to meet women’s needs in ways commensurate with the complexity of issues women were dealing with at any given time. Systems were organized according to rigid structures of limited hours of operation, strict appointment schedules, and an inability to respond to urgent needs as they arose. Systems were not well integrated and consequently women were left navigating multiple and varied services. Additionally, the cultures of such systems were problematic as they perpetuated stigma and discrimination.

The inability to respond to the urgency of women’s needs was a significant challenge. Phrases such as systems *“don’t really understand the acuity level of the community”* [SP04] were common. Furthermore, the lack of accessible information about services available for organizations was equally problematic with women noting that *“you have to dig around”* and *“really go looking”* for information [W16]. This lack of available information can particularly impact women dealing with mental health struggles; W06 stated *“somebody that is in a well-balanced mental state can go online and look reasonably*,* but when you’re desperate and struggling*,* it is not always that easy. It gets really confusing.”* Furthermore, women reported having to display the right *level* of need to be eligible for support, which made it difficult for some women to reach support, even when urgently needed, as W07 described:

*“There is kind of like this very finite realm of people that can be helped by a lot of services … either you’re suffering too much and they go we’re not equipped to handle that…or not suffering enough where they’re like well, you don't sound like you're actually doing that bad.”*

Systems were also not responsive to substance use or mental health crisis situations in particular. SP01 shared that women seeking treatment from detoxification services were unable to access this, even in the longer-term, owing to lengthy waitlists; *“By the time that detox date comes around*,* they [women] don’t want to go anymore … that ship has sailed”*. Further, even when women were able to receive support for such crises at a later date, the delayed responses often worsened the issues that they were facing in the meantime.

Accessibility barriers disproportionately affected women experiencing financial strain. Indeed, many services required phone communication, official identification, capacity to finance services, or independent transportation. These requirements exacerbated the systemic obstacles women faced in accessing or using these resources. In many organizations such as foodbanks for instance, a permanent address and government identification were often required, yet women experiencing food insecurity were those most likely to not have these. Similarly, as W18 noted, the capacity to pay for assistance was especially problematic. This barrier reflected social income assistance rates below a living wage and escalating costs of living:

*“I wanted a counselling service and I had to pay … $50 per session. I only make $733 a month*,* I pay $500 to rent*,* that leaves me with nothing* […]. *It deters you from wanting to get help because it’s not covered and … it’s really hard to come up with the extra money*,* especially when you’re struggling to buy food*.*”*

Waitlist processes further exacerbated the barriers imposed by poverty, including being homeless or precariously housed. Indeed, to be waitlisted and eventually receive services, women were required to be contactable (e.g., have access to a phone, have a permanent address). The inability to be contacted contributed to missing an opportunity for access which further delayed or denied accessibility among women:

*“You lose your phones*,* so you don’t have a phone number where you can call a person one day*,* and then have them call you back.* […] *It’s just ‘oh get back to me tomorrow.’ ‘How am I going to get back to you tomorrow; I might not be at a phone tomorrow. I don’t even know where I’m sleeping tomorrow.”* [W21].

Furthermore, the way in which services were provided often did not align with women’s lives and needs. Typically, only scheduled appointments were offered. The degree of disadvantage women experienced including significant health inequities, poverty and homelessness created chaos for women. Women were often forced to prioritize one need over another, making fixed appointments an untenable option:

*“To keep an appointment*,* you ain’t got no clock*,* you don’t know where you’re sleeping … it is just a spiral down. If you ain’t settled yourself*,* you ain’t getting nothing done. If you ain’t got your head somewhere where you’re sleeping and you can get up and get ready … you’re not getting nothing done the next day. Like how do you be at ease if you’re out trying to find somewhere to sleep?* […] *You’re not thinking about the next day*.*“*[W15].

*“I find that* [substance use needs] *takes up a lot of my day sometimes just trying to get better and the important things like getting to the doctors or getting on safe supply* [drug treatment], *is a harder task.”* [W03].

Providers also recognized that, due to the degree of disadvantage at play in women’s lives, *“general time management*,* to get to a scheduled appointment* [is] *very difficult”* [SP19]. For example, SP04 explained:

[Women] *are … facing so much violence and chaos. Like the amount of times you get to someone’s room for an appointment and maybe things don’t work out. They’re dope sick*,* something’s happened*,* a friend needs something*,* they forgot what day it was and they’re completely unprepared.*

Limited service-hours were additionally problematic, leaving women without support during critical times. As W04 explained *“everything closes at 4:30*,* so then you’re stuck for the night.”* This meant that women were often left with very few options for assistance, if any, during times when they might most need support. Providers from across all three communities recognized the restrictions of service hours as a significant gap in how services are provided for women:

*“A lot of the ladies … that we support that are just looking for clothing*,* mostly ladies that are on the street level at nighttime when every other agency is closed*,* because everything is Monday to Friday 8:30 to 4:30 … That I find really tragic*,* that no one in our communities have stepped up to that weekend or later night when people are hurting still.”* [SP16].

Moreover, services were designed to support a singular focus or issue versus an integrated, holistic approach. The siloed nature of supports failed to address the systemic inequities shaping women’s lives and the interconnected nature of their needs. Women’s needs were compartmentalized within these systems and they had to attempt to fit into service’s specific and limited scope. As W19 explained: *“it’s not like you can just type out on the internet and find an agency that can help one*,* two*,* or three of those things* [housing, financial aid, child custody, health care]. *You have to go through one*,* and they will branch you out to all those other ones.”*

The problematic culture and ethos of services, which can perpetuate stigma and discrimination, presented significant access barriers to health and social care services, often discouraging women from seeking help or returning for continued support. Women reported feeling negated as being worthy with phrases such as: *“they are kind of looking down at you*,* like where they’re up here and you’re down here”* [W16]. Others detailed *specific intersectional* stigmas they faced embedded within racist, sexist, and ableist assumptions (e.g., regarding addiction, Indigenous identity, gender identity). These experiences contributed to women not having the opportunity to complete their care or service, thereby exacerbating inequities in access and receipt of essential health and social care:

*“Being native and being a woman and being overweight and not being able to read very well*,* is quite difficult … there’s times where I have to fill out things*,* and when I go to ask them to help me read something they look at me like I’m stupid … that’s just embarrassing so I just walk out.”* [W02].

Systems whereby women’s self-identified concerns were ignored and paternalistic assumptions about what women required further added to the problematic design and implementation of health and social care services. As W08 noted:

*I’ve had a lot of doctors … tell me that being on medication* [for anxiety, ADHD, and depression] *would be better for me … I am not going to put my body through that because I have already been through that … It’s like no*,* you’re not telling me that this needs to happen … So just understanding that if a person is saying that they don’t want to be on medication…*.

These systemic issues highlight the need for comprehensive reform in service design and delivery to better meet the complex needs of women seeking support.

### Theme two: service providers support and empower women to overcome challenges

Women and service providers detailed specific practices and empowering strategies providers used to help women navigate the challenges faced within the broken systems outlined above. These strategies included: providing key information, and therefore instilling hope; assisting with administrative tasks such as liaising with services and facilitating referrals; providing material resources and reminders; and addressing discrimination through accompaniment and advocacy.

Service providers offered crucial information and options for navigating various systems, even when their specific service or organization could not meet the woman’s present needs. Certainly, they strove to avoid *“leaving somebody hanging high and dry”* [SP02] or allowing for *“a door shut in their* [a woman’s] *face”* [SP01], since this often led to women losing hope regarding receiving support. W19 emphasized the value of this type of support, stating that without her *“heaven sent”* service provider, she *“would not even know what these agencies offer.”*

Recognizing the impact of trauma inclusive of previous derogatory and discriminatory clinical encounters for women’s ability to engage in particular tasks in a given moment (e.g., feeling anxious about calling a service) was important. Consequently, providers often assisted with administrative tasks, such as completing required paperwork/application forms, liaising with agencies on behalf of women, and providing formal referrals. As one service provider explained:

*“I can help them … get their ID that they might need to apply for social assistance*,* help them if they need to provide their banking*,* if they need to provide any sort of documents. I help gather that information for them and submit it and kind of liaise between* [client and services].*”* [SP19].

Women spoke of the positive impact of this support noting that they were not left alone in their attempts to navigate the system and that the support enabled completion of a task necessary to access a specific service.

Providers recognized the importance of a person-centred approach, which they defined as each woman having different strengths and circumstances and tailoring their level of facilitation accordingly. As SP18 described:

*“Some people can just take a phone number and say okay*,* thanks very much; I’m going to call them myself … not everybody is able to do that*,* right*,* whether it’s anxiety or trauma*,* comprehension*,* English as a second language*,* mental health*,* addiction*,* a combination of a whole bunch of stuff … we assess and say okay*,* ‘I think this person needs a more facilitated referral approach to get to that agency that is most appropriate for them.‘”*.

For women dealing with multiple forms of disadvantage, more engaged, formal referrals from service providers were very helpful. For example, W06 explained:

*The counsellor that I had before … she called* [organization] *for me and she got information for me*,* and said … I spoke to this person. So*,* it made it a lot easier for me to make the next call because my counsellor already kind of opened the door for me and gave a little bit of the background history*,* so I didn’t have to start fresh*,* so that made a big difference.*

Providers also helped to remove pressure and potential re-traumatization by facilitating initial requests and taking on organizational tasks so women were *“not bombarded”* and could ultimately *“focus on their healing journeys*” [SP09]. SP07 emphasized the importance of preventing women from having to repeatedly share their experiences:

*“It’s like how can we make the legal system less shitty … how can we approach this in a way that stops re-traumatization*,* because that is what happens when women are made to tell the same story over and over again.”*

To address accessibility and connection barriers, service providers often found ways to provide material resources, such as phones or laptops, and outreach supports. For example, SP16 explained that *“We’re also collecting used unlocked cell phones right now for the ladies who don’t have cell phones*,* I hope to have 300 or 400 of them by April”.*

Providers also offered reminders and ‘nudges’ to women, who because of underlying health issues (e.g., mental health issues, addiction) or crises grappled with short term memory, to support them with attending rigid, scheduled appointments. W15 explained the value of this support:

*“You do need someone to take you by your hand sometimes … If you would have done it*,* you would have done it on your own*,* and some people can’t … but sometimes I just*,* I don’t do it. I need someone to push me and push me or bug me or find me or something*,* because otherwise I’ll just keep falling*,* because there are all kinds of obstacles in your life beyond being homeless.”*

Service providers also assisted women to overcome challenges related to experiencing stigma and discrimination by accompanying women to appointments and advocating on their behalf. As one woman stated the value of her trusted service providers *“just stepping in and talking for me sometimes when I couldn’t”* [W04]. Women described the impact of this support, with some mentioning that they were treated as *“just a number”* or discriminated against prior to receiving support from other service workers:

*“The doctor was not listening to me at the time. … When* [my service provider] *was around … then she treated me like a normal person. But if I was by myself*,* it was … I was a child.”* [W03].

Service providers discussed how through their intervention they could improve how their clients were treated when interacting with different systems:

*“The second you walk in the* [hospital] *door and you fit what somebody might think is a ‘user’*,* immediately the judgement is passed … Luckily with the support of us*,* we kind of change that and make it a little more comfortable.”* [SP01].

Providers also talked about advocacy in relation to systemic challenges such as problematic design, rules, and decisions of various systems (e.g., housing, health, legal, welfare). Most providers acknowledged that these systemic issues created significant challenges for women in accessing supports and there was need to advocate for broader change to these systems. SP03 spoke to holding services accountable for supporting women:

*“I feel like every institution you work with*,* the healthcare system*,* housing system*, [child welfare services] *there’s rules that don’t benefit people*,* they benefit people at the top … They want to control people*,* right? And I always try to push that*,* try to advocate for clients. […] When you try to push that and you try to advocate*,* there’s always friction…*,* You’re just dealing with systems that don’t work … I try to push those boundaries … A lot of the systems they don’t care about people at the end of the day…”*.

Women also demonstrated their strengths, agency, and determination by advocating for themselves, often describing the demanding and distressing process of accessing services as a *‘fight’*. W06 stated, *“If you don’t fight for help*,* then you’re just lost”*, while W09 described her self-advocacy process:

*“The day that I got out of the hospital*,* the next day I started advocating for myself*,* I reached out to services … I have tried everything I can possibly think of and I think I have exhausted every resource in this community*,* but wait times are impossible and I still haven’t figured anything out*,* other than the small little things…”*.

### Theme three: building an effective working relationship

Women emphasized the importance of cultivating effective working relationships between themselves and service providers, which was crucial for engaging with services and overall well-being. Trust was central to these relationships, which was built over time through informal interactions and meeting women *“where they are at”*, literally (e.g., through outreach), and figuratively (e.g., by approach without judgment). It was also important that service providers took an individualized, person-centered approach and worked collaboratively with women. Despite existing constraints, service providers strived to deliver ongoing support.

Women discussed their relationships with providers, describing their bonds as ‘*real*’ and *‘meaningful’*, with one woman going so far as to say that her worker was her *‘soul’* and ‘*rock’* [W21]. Providers were seen as needing to fulfill multiple roles, including *‘stable friend*’, ‘*mom*’, *‘sister’*, and *‘cheerleader*.*’* Both women and providers highlighted that trust was crucial to an effective working relationship, and while this took time to build, it was clear that *“things cannot move forward without trust in the relationship”* [SP05]. Indeed, establishing a sense of trust was crucial for creating a safe space for women to be able to engage with service providers as this allowed these women to feel comfortable sharing their stories and openly discussing their needs. As W10 explained:

*“To go through these experiences*,* it’s hard to share. It’s very personal information so you really have to make somebody feel safe and there’s only time that can build up that trust unfortunately which my case worker was able to do with me over a few phone calls. Her questions weren’t*,* there were very forward*,* but some of them she knew to ask a little bit later.”*

Providers built rapport and trust with women through informal engagements such as sharing meals, going for walks, offering safe spaces to vent, or providing supplies (e.g., food, clothes). SP03 noted: *“If they want to go out for ice cream and not do anything*,* it’s just building that relationship and building trust*.*”* Though some service providers acknowledged that others might view less formal engagements as unprofessional or over-stepping boundaries, they felt that such engagements were uniquely appropriate when supporting women with complex needs. As women shared a common experience of gender-based violence and discrimination, trust was reported as difficult and time spent was crucial to overcome distrust. As SP03 went on to explain *“it gets you further. You create more meaningful change*,* you’re able to build better relationships*,* they trust you faster.”* Certainly, women valued this type of relationship building and could quickly tell when providers *“are actually invested in my life becoming better”* [W07] through their approach to communication and engagement.

Service providers also *“met women where they were at”*, which involved both physical outreach and a non-judgmental approach. SP04 explained that outreach was a *“huge piece”* of her work:

*“In terms of getting to see my clients*,* I go to their housing*,* I meet them where they’re at. For some of my folks who are harder … I’m going to walk around and check out your usual haunts and see if I can find you and connect*,* and* [see] *what can we get going. But outreach is huge.”*

Providers also spoke about meeting people where they were at by taking a non-judgmental stance. Indeed, they worked with women regardless of their circumstances and *“created a space of nonjudgment and safety”* [SP15]. For instance, SP09 described supporting women in abusive relationships who did not want to leave their partners, without judgement regarding this:

*“I say to clients like*,* even if it’s a domestic violence file and you’re telling me that you love this partner and then in two weeks you come back and you tell me you’re back with them*,* fine*,* that’s okay.* […] *I’m going to help you make informed choices and my door will always be open. So even if you go back a hundred times*,* you can come back here at the end of the day*,* we can talk*,* we can talk about safety planning and making sure that you’re keeping yourself as safe as you can.”*

Furthermore, providers assured women that they would continue to offer support regardless of challenges they might face or behaviours they might exhibit. SP19 spoke about continuing to support women who were *“cursing”*,* “swearing”* and *“name calling”* during engagements, recognizing that this might be related to substance use, mental illness or external stressors.

Service providers also took an individualized, person-centered and collaborative approach to engagement, prioritizing women’s self-identified goals. They emphasized women’s strengths and saw them as experts on their situations, thus they ensured that women took a central role in decision-making. SP14 emphasized:

*“Autonomy is a big piece … We fully believe that the clients that we have are experts in their lives and they can keep themselves safe and that they have all the tools and strengths necessary … make those necessary changes*,* and it is just a matter of kind of walking alongside them*,* not directing them*.*”*

Women directed discussions regarding identifying their needs and goals, rather than goals being imposed on them, as SP03 explained:

*“It’s not about…*,* me thinking that this person needs this and me forcing that onto them … a lot of the girls are homeless and I could think oh they need somewhere to be or they need a shelter*,* right? But when you talk to them*,* a lot of them are like ‘I don’t want a shelter*,* I feel safer being outside.‘”*.

Flexibility in the types of programs offered was also crucial. W09 noted: *“there is a whole array of services … I can kind of pick and choose*,* right? So that’s a strength right because that gives people the freedom to shop around a little bit*.*”* Furthermore, it was also important that women were offered choices about the ways in which support was provided, including when and where engagements took place, rather than requiring them to follow rigidly defined programs. This was especially important when women had other priorities to manage, as SP07 explains:

*I still say … is this still an okay time with you? When it is not*,* client’s feel*, [gasps] *‘but*,* I want you to know I care and respect*,*’ and it’s like … you’re a single parent nine times out of ten and you’ve got a lot of things*,* like you’re trying to live your life*,* I might be an important*,* but I am a very tiny part of your life; you can rebook all day every day.*

Beyond this, women played a significant role in determining the course of action required to meet their goals. Indeed, they were able to define ‘success’ in their own way. SP05 stated: *“what’s important is that a woman is moving forward and that is coming from a place that she believes she is; not necessarily because we think she is.”* Women also made various key decisions, such as whether or not to report GBV to the police, or whether they wished to engage in counselling, if and when such opportunities were available.

Finally, providing consistent and ongoing support, and sustaining the possibility for re-engagement with services, was important for maintaining an effective working relationship. Women appreciated this steady support: *“I’m very appreciative of the fact that it’s easy to get hold of somebody and to be able to just reach out and go ‘hey*,* help*,* please’”* [W03]. Service providers spoke about the importance of ‘keeping cases open’, especially in the context of women experiencing IPV where the need for re-engagement might emerge suddenly. For example, a woman who makes the decision to leave their abusive partner might be seeking support in a number of areas at the time at which they leave (e.g., housing, financial support). SP13 spoke to this, noting that women might experience financial constraints in an abusive relationship such that *“when they leave*,* they literally have nothing”* and therefore require supports quickly in several areas. Although the importance of ongoing support was clear, service providers experienced a tension between wanting to keep cases ‘open’ to encourage re-engagement at any point and the competing need to *“make room for other women”* [SP01] waiting to access services.

## Discussion

Our findings illuminate critical public health challenges faced by women experiencing GBV as they navigate fragmented health and social services across multiple siloed systems. This fragmentation emerged as a pervasive issue across all three Canadian cities studied, forcing women to navigate a complex and often disconnected web of supports. The women we interviewed were navigating diverse health and well-being trajectories, that were directly impacted by structural disadvantage that ranged from homelessness, poverty, gaps in accessible and appropriate health care and ongoing GBV in their lives. Within this context, our findings show that the systems within which health and social services are organized, are not designed to meet women’s complex needs but instead, often perpetuate and replicate the systemic inequities they face. Specifically, flaws in the organization and delivery of services illustrated how these systems are not responsive to crisis situations or the structural disadvantage influencing women’s lives (i.e., poverty); systems were inflexible, resource limited, and siloed, thereby structuring services in ways that make access challenging for those who need them most; and systems with comprising problematic cultures perpetuate stigma and discrimination. This underscores the need for a coordinated, cross-sectoral approach to addressing GBV and its health consequences.

From women’s accounts, and echoed by service providers, findings illustrate specific practices and empowering strategies which providers used to support women while working within these broken systems. These included providing information that instills hope at the outset; completing administrative tasks, liaising, and facilitating referrals to help with initial connection; offering material resources and ‘reminders’ to increase engagement; and redressing discrimination and stigma through accompaniment and advocacy. Beyond this, the importance of building an effective working relationship between women and service providers was paramount. This relationship was characterized by trust, rapport, safety, non-judgment and collaboration, and cultivated through informal interactions: engaging in outreach and meeting women where they are at; taking an individualized, person-centered approach; providing opportunities for autonomy and power sharing; respecting different needs, goals, and definitions of success; and facilitating ongoing, consistent support with ease of reengagement. Thus, we saw that as systems failed to meet the needs of women, certain community-based services, and providers working within them, were forced to compensate. From a public health standpoint, these strategies represent important efforts to bridge gaps in fragmented services and provide more comprehensive care.

Our findings show that systems often put the onus on women to have specific resources (e.g., phones, permanent address, formal identification, financial means, or independent transportation) in order to enroll in and access their various services. Such requirements contradict the very purpose of having services, especially services that are meant to support those women dealing with chronic or episodic poverty. Indeed, low-barrier, flexible services that allow women to set goals and focus on attending to fundamental needs (e.g., food, housing, safety) are crucial in these circumstances. Moreover, research shows that in cases of IPV one of the key barriers to leaving an abusive relationship is lack of material resources – for instance, women are often placed in circumstances where they are financially dependent on their partner, and lack alternative options for shelter, employment, income, and childcare [57, 58]. Moreover, demands for resources can also create additional delays in service access (e.g., when women fall through cracks of a waiting list because they do not have reliable contact information) which is not only significant in emergency cases but has also been found to impact outcomes of mental health or substance use programs, as client desire/motivation for such supports are time-sensitive and can change significantly over time [59]. In light of this, there is an overarching need to address the material conditions of poverty that women are subjected to (e.g., providing access to safe, affordable housing, financial and food security) thereby putting the onus for providing the resources necessary for accessing services back on the systems.

The siloed systems were often unavailable, and difficult to engage with, which as demonstrated elsewhere, regularly results in women’s needs being compartmentalized and the interrelationship between their needs and structural disadvantage being overlooked [2, 60, 61]. Similarly, our results showed a lack of holistic supports, with services being designed to support one issue at a time and on a set schedule that created further barriers to access. Indeed, women experiencing GBV are often balancing a variety of competing priorities including: safety, housing, food and financial security, childcare, mental, physical and sexual health, employment, and broader well-being [62, 63]. All of this adds to the need to honor the numerous and interrelated issues women must deal with and provide services in a flexible manner. Flexibility in service provision has been recognized as a key component to providing effective care for women experiencing violence and has long since been recognized as crucial for the provision of care for those who are street-based or living in poverty [2, 37, 47, 64, 65]. The initial success of integrated care models (e.g., The Pink Code Pathway; [66]) in coordinating medical, psychological, social and legal support under one comprehensive framework suggests a promising direction for addressing these complex challenges.

Given that head injuries and probable traumatic brain injury are prevalent among women experiencing violence, including IPV, the impacts of these injuries must be considered when providing health and social services for women [67, 68]. Indeed, studies on women survivors of IPV have found that the occurrence of potential brain injuries varies widely, with estimates ranging from 19 to 100% depending on sample characteristics [69]. Traumatic brain injuries can impair memory, attention, and concentration, potentially hindering a woman’s ability to consistently engage with support services [70, 71]. In this context, the role of service providers in offering reminders and “nudges” becomes crucial. Such support can help compensate for trauma-related memory difficulties, ensuring women maintain engagement with necessary services. Moreover, providing clear and easy to understand information about relevant services and ensuring that different options for support options are available are also crucial. This approach not only addresses practical needs but also contributes to restoring a sense of control and agency, which is often compromised in traumatic experiences [2].

Our findings also speak to the importance of cultivating an effective working relationship to provide services for women experiencing GBV. Specifically, this approach focuses on taking care and time to establish rapport and trust within the client and service provider relationship. Building rapport by improving relationship quality through personal connection and enjoyable interactions, is fundamental to any client and service provider interaction as it enables open communication and information sharing and allows providers to make better clinical decisions [72, 73]. Trust with services can be difficult to establish for women who are experiencing GBV, many of whom may have had their trust eroded through experiences of abuse and negative encounters with service providers [74–76]. Yet patient trust in healthcare providers has been shown to correlate to better health outcomes and service utilization [77]. Indeed, trust has been identified as an essential facilitator of collaboration and partnership for women and service providers throughout the literature [65]. Providers can establish trust with their clients through effective communication (e.g., active listening, providing information), showing that they care about their patients (e.g., respecting their individual experiences, being committed to solving their health problems), and exhibiting competence (e.g., being knowledgeable) [77]. However, our findings reinforce that establishing rapport and trust with women experiencing GBV meant engaging in ‘less formal’ interactions that some might view as unprofessional or over-stepping boundaries, yet were affective strategies to engage with these women. Such tensions around the appropriateness of using informal strategies to establish rapport and trust in practice are common across a variety of different care fields (e.g., nursing, psychology, social work) [78–81]. Moreover, though we heard from both women and service provider participants that secure trust took time and continuity to build, given that women often dealt with crisis situations, lessons learned from the literature on building ‘swift trust’ [82, 83] rapidly in time-sensitive situations may be particularly relevant for providers. This form of trust, may allow for immediate action and support in urgent cases and leave space for secure trust to be developed down the line [84].

This paper has some limitations that need to be acknowledged. First, the study participants were predominantly cisgender and White, potentially underrepresenting the experiences of gender diverse people and racialized women. Future studies should employ targeted recruitment strategies, such as partnering with organizations serving gender diverse individuals and racialized individuals, to ensure a more representative sample and a better understanding of intersectional impacts on service access. Second the data collection occurred post lockdown during the COVID-19 pandemic, which may have influenced participants’ experiences and perceptions of service access and delivery. Third the sample sizes in each site may be considered small (approx. 15 participants per site), yet the strength of our sample lies in its composition and geographical diversity. By including both service users and providers, we were able to gain a more comprehensive understanding of the service landscape and associated challenges. Additionally, by focusing on three Canadian cities of varying sizes and local contexts, we enhanced the transferability of our findings. This approach allowed us to capture a nuanced view of the issues at hand, identifying both common themes and context-specific factors that influence service provision and utilization for women.

## Conclusion

Our findings show women’s strengths and agency in navigating within oppressive, fragmented systems not designed to meet their complex health and social care needs. We also heard about how service providers went above and beyond in finding practical ways to support and empower women to overcome challenges to engage with services and attempt to meet their needs. However, despite how hard women and service providers worked, they were essentially operating within broken, fragmented, systems, that were not well designed to foster women’s success. All women have the right to live their lives free from interpersonal and systemic violence. Facilitating safe access for women to responsive, holistic health and social services that take into consideration how women want to access and engage with supports is a crucial first step. Our research demonstrates that women’s agency can best be supported, and their complex and inter-related needs can best be addressed, by services that are provided in the context of an effective working relationship built on trust, safety, respect, collaboration, power-sharing, and understanding.

Though recognizing the strengths possessed by women and service providers is important, we must take care not to place the onus for improvement and sustainable change on these individuals, as this could set a dangerous precedent. Rather, we wish to acknowledge that the challenges to accessing services facing women experiencing GBV are systemic in nature and cannot be overcome without changes to the broader systems (e.g., health, housing, criminal/legal, social welfare, labour, political) that perpetuate them. This highlights the urgent need for systemic change to address the fragmentation of services and create more integrated, responsive support systems for women experiencing GBV. Additionally, it is essential that these system-level changes be accompanied by societal action aimed at disrupting patriarchal and sexist norms that create, sustain and reproduce GBV [51–53]. Addressing these systemic issues is not only a matter of social justice but also a critical public health imperative, essential for improving population health outcomes and reducing health inequities related to GBV.

## Electronic supplementary material

Below is the link to the electronic supplementary material.

Supplementary Material 1

## Data Availability

The datasets generated and analyzed for this study are not publicly available due to issues of safety, sensitivity and privacy. Participants in this study consented only to sharing of their research data within the immediate team and not with external parties. Public deposition would breach compliance with the protocols approved by our research ethics boards. Inquiries about access to data should be directed to the Principal Investigator (Vicky Bungay).

## Footnotes

1 We use the terms woman/women throughout this paper as inclusive of anyone who identifies as a woman.
