# Supplementary material for: Navigating fragmented services: a gender-based violence (GBV) critical feminist analysis of women’s experiences engaging with health and social supports in three Canadian cities
Source: BMC Public Health. 2025 Mar 31;25:1213. doi: 10.1186/s12889-025-21919-w (PMC11956248; doi:10.1186/s12889-025-21919-w)
Supplement: Supplementary file 1 — Supplementary Material 1 [file 12889_2025_21919_MOESM1_ESM.docx]

**Scaling up Trauma and Violence Informed Outreach with Women Affected by Violence – Interview Guides**

*The following interview guides were developed for the sole purpose of the study, funded by Social Science & Humanities Research Council (Scaling up Trauma and Violence Informed Outreach with Women Affected by Violence, #895-2019-1001). The interview guides cannot be used in part or entirety without permission from the lead investigator, V.Bungay.*

**Interview Guide – Participant Group: Women**

# Introductory Script

Thank you for taking the time to meet with me and agreeing to this interview. When we talked through the consent process, I described that we are doing this research to better understand how to meet the needs of women who live in [your community] and what types of services are working and now working that can help to meet women’s needs.

We appreciate that you have engaged with services in this community. Therefore, we are specifically interested in knowing what it’s been like for you to engage with the services offered here, including for example [name of partner organization]. We also want to know, from your experience, what you think worked well for you and what, if any, recommendations you might have about how services could do things differently to meet your needs.

1. To get started, it would be helpful if you could tell me a little bit about yourself. There are just a few questions
   1. In what year were you born?
   2. What is your highest level of education?
   3. On a scale of 1 – 10, with 1 being easy and 10 being impossible, how difficult is it for you to live on your income right now?
   4. In what country were you born?
   5. How do you define your ethnicity?
   6. Do you have a regular health care provider?
   7. Do you have a regular support worker?
2. Thank you for answering those questions. It would be helpful now to move on to discuss your experiences with accessing services in this community. A good starting place is if you could tell me about the types of services you are currently using.

Probes:

1. Some people find it helpful to think about the first time they learned that this program or service existed. Can you tell me more about that please? How did you learn about the service? What brought you to this service?
2. What happened when you first engaged with the service? What was it like?
3. [*if there was a referral*] who made the referral?
4. [*if someone introduced them*] how did that introduction happen?
5. Over time, have you continued to work with this service? Please describe why or why not?
6. Please tell me about how you decided to work with the [service]? For example, tell me about a situation where you thought that accessing this service might be helpful for you.

Probes:

1. Please elaborate on what you hoped they might be able to help you with.
2. How long did you know them before you decided that they could support you in some way?

1. What sorts of things were you thinking about when you decided to engage with them to support you in some way?
2. Please describe for me what you think were/are the most positive things about working with the [organization] for you?
3. Please describe any barriers you experienced in trying to receive services with [organization]
4. Have you had a situation where you are working with more than one service provider or organization at once? If so, please describe for me what that was like for you?

Probes:

1. Describe if and how information was shared between these different people or organizations?
2. What sorts of things did you find helpful for you in working with more than one person or organization? What sorts of things were challenging?
3. What do you think are some of the benefits you have received by participating in this program?
4. How supported did you feel working with the outreach team?
5. Overall, what do you think about the way services are provided to women in your community.

Probes:

For instance:

- - 1. What do you think are some of the biggest strengths of the services and programs you have accessed?
    2. What do you think are some of the biggest problems or challenges with the services and programs you have accessed?
    3. What services do you think are missing in your community?
    4. What do you think service providers need to know about working with women in your community in order to be able to do their job well?

1. What other thoughts or ideas do you have about services for women in this community that we have not discussed? For example, is there anything else you think we should know about to better support women to engage with services in your community?

THANK YOU

# **Interview Guide – Participant Group: Service Providers**

Thank you for taking the time to talk to me today about the services provided by your organization and your experiences of service delivery with women in your community. As you know, we are particularly interested in service delivery with women who are regularly underserved in existing services and often experience barriers to services associated with challenges in their everyday lives such as competing needs and demands around housing and food security and attending to health care, and perhaps previous challenges in accessing services.

1. To get us started, please tell me a bit about yourself and your role at [organization].

Probes:

- How long have you worked at [organization]?
- Tell me your actual job title and describe for me what that means in terms of what you do in your work?
- What sort of education or training do you have for this job?
- What other types of work have you done?

1. It would be helpful to hear more about your organization. Please describe for me the types of services your organization provides.

Probes:

Please describe:

- your organizational mandate,
- range of services offered at organization,
- how your team works together to provide services,
- For decision makers: staff complement and funding sources.

1. As we are concerned with women’s services, please tell me about who your organization serves. For example, describe for me the range of clients that you serve. It may be helpful to consider such things as: client demographic characteristics (e.g., age, housing status) and common needs.
2. Please describe for me how women access your organization. For example, are women referred, do drop-in, perhaps both?

Probes:

- If women do drop in, how do they learn about your organization? How effective do you think this process is?
- If you receive referrals, describe that process for me. How effective is this process?

1. Please describe how you (service provider) or your (decision maker) organization work with other organizations and services in your community. For example, what other services and programs do you work with to meet the needs of your clients?

Probes:

Please describe:

- what kinds of services they offer,
- who they interact with within their organization (clients/patients, colleagues, and other organizational stakeholders),
- and who they interact with outside of their organization (e.g., health care and service providers at other organizations, police, etc.).
- How successful are these working relationships in meeting women’s needs? Why or why not?

1. Please describe for me what you think some of the challenges and strengths are for your organization and others in this community in providing services to women.

Probes:

Please describe:

- What you think about this community and the needs of women;
- If there are any specialized or targeted services that are particularly helpful for women you work with?
- If there are any specialized or targeted services that are needed but not available or difficult to for women to access?
- How effective are the current services in your community in meeting women’s needs? Please explain why or why not?

1. *For organizations that do not have outreach as an element of their service delivery model:* As this study will eventually be implementing an outreach model of service delivery, please describe what role you think outreach has in supporting women in your community.

Probes:

- What does outreach mean to you?
- What do you think are some of the benefits and limitations of outreach as a strategy for connecting with women?
  1. Do you have experience collaborating with outreach workers? If so, what has that been like? For example, what are the referral procedures used between your agency and outreach? Are there any particular outreach teams you work with?
  2. Do you have any recommendations for working in better ways with outreach workers and if so, please describe.

1. *For organizations that have outreach as an element of their service delivery model*.
   1. Please describe what role you think outreach has in supporting the work of health care and service providers in traditional settings (e.g., clinic or agency). For example, describe the working relationships with health care and service providers in your organization and outside your organization. What are the referral procedures?
   2. Please describe any recommendations you might have for how providers in traditional roles can better utilize outreach workers.
2. Is there anything else you would like to share with us about services for women in your community.
3. Finally, can you please answer the following questions about yourself. These details are confidential but help us to provide a description of the group of people who took part in the study.

What is your current age? _________________

What is your highest level of education? ______________________

Do you hold a professional licensure (e.g., RN, Registered Social Worker, Nurse Practitioner)?

No _____________

Yes: __________ describe ____________________________

How long have you been in your current role? _________________________

How long have you worked within the [name] community? ___________________

How do you describe your gender? ___________________________
